# Multidimensional roles of cfDNA fragmentomics in preeclampsia: from placental hypoxia and TLR9 inflammation to clinical risk stratification

**DOI:** 10.3389/fmed.2025.1539651

**Published:** 2025-09-19

**Authors:** Ziyi Guo, Bin Zhang, Di Yang, Li Wang

**Affiliations:** Changzhou Maternal and Child Health Care Hospital, Changzhou Medical Center, Nanjing Medical University (Changzhou Maternal and Child Health Care Hospital), Changzhou, China

**Keywords:** cell-free DNA, preeclampsia, placenta, fragmentomics, methylation

## Abstract

Cell-free DNA (cfDNA) has emerged as a pivotal biomarker for predicting preeclampsia (PE), a multisystem syndrome characterized by placental hypoperfusion and systemic inflammation. This review synthesizes critical advances in the field, highlighting quantitative alterations in cfDNA, fragmentomic profiles, and placenta-specific methylation patterns (e.g., RASSF1A) that demonstrate significant value for early prediction and severity stratification of PE. Mechanistically, placental hypoxia-induced trophoblast apoptosis (releasing cfDNA), epigenetic dysregulation activating TLR9/NF-κB inflammatory pathways, and oxidative stress-mediated mitochondrial cfDNA fragmentation collectively drive disease progression. In clinical translation, integrating cfDNA with complementary biomarkers enhances predictive performance, though limitations persist regarding preanalytical variability and dynamic gestational changes. Future efforts must advance fragmentomics-integrated multi-omics frameworks for precision prediction, where assay standardization constitutes the fundamental translational bottleneck.

## 1 Introduction

Preeclampsia (PE) is an autoimmune disorder characterized by hypertension during pregnancy (1), with an incidence ranging from 3% to 7% in primiparous women and 1% to 3% in multiparous women. Elevated levels of fetal DNA and RNA derived from the placenta have been observed in pregnant women with PE, indicating placental dysfunction, which likely plays a central role in the pathophysiology of the disease. Common pathological findings in preeclamptic placentas include arteriosclerosis, hypertensive narrowing of arteries and small arterioles, fibrin deposition, and infarction, all of which are consistent with inadequate placental perfusion and ischemia and seem to correlate with the severity of PE. In mothers, PE may lead to premature cardiovascular disease in later life, while children born from preeclamptic pregnancies tend to be relatively small at birth, with an increased risk of stroke, coronary heart disease, and metabolic syndrome in adulthood (2). The development of clinical symptoms of PE is thought to result from impaired trophoblastic invasion of maternal spiral arteries, leading to placental hypoxia and the release of inflammatory cytokines, which alter maternal systemic endothelial function, causing widespread endothelial cell dysfunction (3).

Circulating cell-free DNA (cfDNA) refers to trace amounts of endogenous and exogenous DNA fragments present in the bloodstream, existing outside of cells. Apoptosis is generally considered the primary source of cfDNA in serum or plasma, as dead cells are phagocytized by macrophages, releasing digested DNA into circulation to form cfDNA. Gel electrophoresis analysis of cfDNA typically shows a predominant band around 180 bp, with another distribution near 360 bp, which corresponds to the lengths of DNA wrapped around mononucleosomes and dinucleosomes, further indicating that cfDNA originates from apoptosis (4). In contrast, DNA released from necrotic cells tends to be larger, ~10,000 bp, due to incomplete and non-specific digestion. Additionally, the observed increase in plasma cfDNA during hepatocyte apoptosis in mice supports the notion that apoptosis is a source of cfDNA. Notably, the concentration and composition of cfDNA is a dynamic process; cfDNA is protein-bound with a relatively short half-life, with reported half-lives ranging from a few minutes to 2 h (5).

Recent breakthroughs in cfDNA detection technologies—particularly high-throughput sequencing (NGS) and epigenetic marker analyses (e.g., methylation profiling, fragmentomics)—have substantially expanded their applications in non-invasive prenatal testing (NIPT) and early cancer screening (6). Nevertheless, within the preeclampsia (PE) field, although cfDNA's biological features (such as placenta-specific methylation patterns and fragment size distributions) have been robustly associated with placental pathology, their translational potential for early clinical prediction remains systematically underexplored. Current clinical practice relies on maternal risk factors (e.g., hypertension history, BMI) combined with uterine artery Doppler ultrasonography for PE risk assessment; however, these methods exhibit limited sensitivity/specificity and demonstrate inadequate reliability for identifying high-risk populations during early gestation. While established biochemical markers (e.g., PlGF, sFlt-1) provide diagnostic value, their late detection window (typically post-second trimester) fails to meet the critical time requirement for early intervention.

This review synthesizes cutting-edge advances in cfDNA research to systematically evaluate its potential as a non-invasive biomarker for early PE detection through three dimensions: Elucidating molecular mechanisms governing placental cfDNA release and its pathophysiological links to PE; Critically assessing innovative cfDNA detection technologies for enhancing early diagnostic accuracy; Analyzing current translational research bottlenecks (e.g., sample heterogeneity, standardization gaps) and multi-omics integration strategies. By defining the pivotal role of cfDNA in PE predictive modeling, this work will establish a theoretical foundation for developing highly sensitive, first-trimester-compatible non-invasive screening tools. Such advancements are positioned to address critical voids in existing clinical surveillance systems, ultimately improving long-term maternal and neonatal outcomes.

## 2 Relationship between cfDNA and the severity of preeclampsia

### 2.1 Molecular characteristics of cfDNA relevant to preeclampsia prediction

In 1997, Lo et al. first discovered the presence of cfDNA in maternal plasma and serum, introducing the unique possibility of using non-invasive methods (i.e., routine blood tests) to obtain fetal material. Since then, numerous studies have focused on identifying the source, characteristics, and predictive potential of cfDNA, with particular emphasis on predicting pregnancy-related complications and prenatal diagnosis or screening of genetic fetal disorders. To date, cfDNA has been widely used in prenatal screening for aneuploidy, single-gene disorders, chromosomal abnormalities, placenta-associated diseases, and Rh factor assessment (7, 8). Some studies have found elevated levels of cfDNA in the blood of women with preeclampsia (9, 10), but the role of cfDNA as a reliable predictor of preeclampsia remains controversial (11). As such, the predictive value of cfDNA in preeclampsia is still under investigation. In addition to the well-established parameters such as fetal fraction and total cfDNA concentration, increasing attention has been directed toward cfDNA-associated mutational and fragmentation patterns. Mutations refer to specific genetic alterations present in fetal or maternal DNA. For instance, if DNA released from the placenta harbors mutations in certain genes, this may indicate placental dysfunction and has been associated with preeclampsia (PE). Some studies have reported that epigenetic markers, such as DNA methylation, can be utilized to differentiate fetal from maternal cfDNA or to detect alterations in specific methylation patterns. Placental dysfunction in preeclampsia has been linked to aberrant methylation in genes such as RASSF1A, HLTF, and TIMP3. Therefore, assessing methylation levels of placenta-derived cfDNA in maternal plasma may serve as an indirect indicator of placental health (12). Furthermore, the presence of other genetic variations, such as single nucleotide polymorphisms (SNPs) or copy number variations (CNVs), may also be associated with an increased risk of preeclampsia. Certain SNPs have been implicated in elevated PE susceptibility, and the detection of these variants via cfDNA analysis could aid in early risk prediction.

Fragmentation patterns, particularly the size distribution of cfDNA fragments, are another area of growing interest. In healthy pregnancies, fetal cfDNA fragments are typically shorter than maternal fragments, with a predominant size around 166 base pairs (bp). Preeclampsia may result in altered placental DNA release, leading to shifts in cfDNA fragment size distributions, such as an increased proportion of shorter fragments (< 150 bp) (13). This phenomenon is potentially linked to shifts in apoptotic or necrotic processes due to placental ischemia. Moreover, nucleosome positioning patterns may also be disrupted. Aberrant apoptosis in the preeclamptic placenta can modify nucleosome occupancy, leading to specific fragment end preferences, such as an increased presence of fragment ends in open chromatin or gene regulatory regions. These alterations are potentially associated with altered nuclease activity, particularly that of enzymes like DNASE1L3 (14). Additionally, genomic instability, including microdeletions or duplications in certain chromosomal regions, may be more prevalent in preeclampsia and can be detected through cfDNA sequencing. Finally, analysis of cfDNA end motifs—the specific nucleotide sequences at the fragment ends—may reveal disease-specific patterns. Differences in end motif distributions in PE patients could reflect alterations in nuclease activity or underlying cell death mechanisms.

Researchers have measured circulating DNA levels in samples from women with preeclampsia and those with normal pregnancies using real-time polymerase chain reaction (PCR), and the results suggest that elevated fetal DNA levels are associated with preeclampsia (9). Another study reported a fivefold increase in fetal cfDNA levels in symptomatic preeclamptic women compared to asymptomatic preeclamptic women (15). Similarly, it has been reported that fetal DNA levels rise in women who eventually develop preeclampsia, with the important finding that cfDNA levels increase prior to the onset of clinical symptoms (16).

### 2.2 Dynamics of cffDNA and its contribution to overall cfDNA concentration

Circulating cell-free DNA (cfDNA) refers to fragmented DNA molecules present in the bloodstream, with the fetal-derived fraction known as cell-free fetal DNA (cffDNA), which predominantly circulates in the maternal peripheral blood. cffDNA primarily originates from the programmed cell death (apoptosis) or necrosis of placental syncytiotrophoblasts and exhibits placental specificity. Its levels are closely associated with placental function, and significant elevations have been observed in pathological conditions such as placental abruption and preeclampsia (17). During its release, cffDNA exhibits characteristic fragmentation, with an average fragment size of ~143 base pairs (bp), which is markedly shorter than that of maternal cfDNA (~166 bp). This size difference may be attributed to placenta-specific nuclease activity. The release rate of cffDNA is influenced by changes in placental metabolism, and increased release is observed with placental aging during late pregnancy. cffDNA has a very short half-life in maternal circulation, ranging from ~15 min to 2 h, and is rapidly cleared through renal filtration, hepatic and splenic uptake, and nuclease-mediated degradation. This rapid turnover allows cffDNA levels to reflect the real-time status of the placenta; however, it also necessitates prompt sample processing after collection to ensure the accuracy of downstream analyses.

The proportion of cffDNA within the total cfDNA varies significantly with gestational age. In early pregnancy (10–20 weeks of gestation), cffDNA accounts for ~10%−15% of maternal plasma cfDNA, during which the total maternal cfDNA concentration is relatively low (around 50–100 ng/ml of plasma). During the mid-gestation period (20–30 weeks), the proportion of cffDNA increases to 15%−30%, accompanied by a slight rise in total maternal cfDNA due to increased blood volume (18). In late pregnancy (>30 weeks), the cffDNA fraction can exceed 30%, although substantial interindividual variability exists, influenced by factors such as maternal body mass index (BMI) and placental function. For example, in obese pregnant women, expanded blood volume may lead to a relative reduction in the proportion of cffDNA. In pathological conditions such as preeclampsia and placenta accreta, cffDNA levels can be abnormally elevated. Additionally, fetal chromosomal abnormalities, such as trisomy 21, may also result in increased cffDNA release.

### 2.3 The potential of cfDNA as a biomarker for preeclampsia severity

In preeclampsia, the increase in circulating cell-free DNA (cfDNA) levels is associated with markers of disease severity, including preterm birth and worsening systolic blood pressure—two recognized key indicators of disease severity (19). These findings may suggest an increase in cfDNA release following maternal tissue injury (20). Given the close correlation between total cfDNA concentration and preeclampsia, one study analyzed the relationship between total cfDNA levels in preeclamptic patients and surrogate markers of disease severity. The results indicated that cfDNA concentration was negatively correlated with gestational age at delivery and moderately positively correlated with maximum systolic blood pressure (21). Furthermore, the study found that the fraction of placental-derived cfDNA was moderately negatively correlated with maximum systolic blood pressure, but showed no association with maximum diastolic blood pressure.

Fetal sex is considered an important risk factor for preeclampsia, with growing speculation that placental formation and maternal adaptation to pregnancy may be influenced by fetal sex. Therefore, it is essential to characterize fetal sex when studying the pathophysiological processes at the maternal-fetal interface (22, 23). Previous studies have indicated that the amount of placental-derived cfDNA in maternal plasma is higher in cases of preeclampsia; however, these studies were limited to male fetuses and employed alternative methods (such as PCR for Y-chromosome material), making it difficult to ascertain the specific contributions. In healthy non-pregnant individuals, cfDNA primarily originates from hematopoietic lineages, with smaller contributions from endothelial cells, neurons, and hepatocytes. Understanding the exact sources of maternal cfDNA can enhance insights into the pathophysiology of preeclampsia and determine whether cfDNA sources are related to disease phenotypes, given the particularly heterogeneous manifestations of preeclampsia (21). In addition to increased production, cfDNA levels are also influenced by clearance mechanisms found in the liver, spleen, and kidneys. It has been proposed that placental cfDNA increases 3 weeks prior to the clinical manifestations of preeclampsia, a rise attributed to accelerated apoptosis of trophoblasts.

In a large multicenter study involving 44 women with preeclampsia and 53 controls, it was concluded that not only are fetal cfDNA levels elevated in preeclampsia, but maternal cfDNA levels also exhibit a similar increase, both showing a tenfold rise compared to the control group. Moreover, this study's population was significantly larger than those previously described, allowing for stratification of preeclampsia cases based on the severity of symptoms. The analysis included three cases of HELLP syndrome (hemolysis, elevated liver enzymes, low platelet count) and four cases of eclampsia, revealing that the increases in cell-free fetal and maternal cfDNA corresponded with disease severity. Notably, cfDNA levels in women with severe preeclampsia increased by 3.5 times compared to those with mild preeclampsia, and were tenfold higher than in the control group. Furthermore, it was demonstrated that the levels of these two types of cfDNA are correlated in pregnancies affected by preeclampsia, unlike in normal pregnancies. Recent observational reports on cell-free fetal DNA and maternal DNA levels in pregnancies with preeclampsia and subsequent HELLP syndrome have yielded similar findings. These authors also noted that, compared to women with mild preeclampsia, levels of cell-free fetal and maternal DNA in pregnancies complicated by HELLP syndrome nearly quadrupled (24). Maternal cfDNA levels were similarly elevated. In summary, the increase in disease severity is associated with enhanced release of cell-free fetal and maternal DNA into the maternal circulation. These studies collectively suggest that quantitative and fragmentomic profiles of cfDNA are significantly associated with the clinical stratification of preeclampsia. Taken together, these findings indicate that cfDNA levels, particularly the placental-derived fraction, are closely associated with the severity of PE. This suggests that cfDNA may not only serve as a biomarker of disease severity but could also potentially participate directly in the pathophysiology of PE by mediating immune-inflammatory responses. Key evidence and correlations are summarized in Table 1.

**Table 1:** Summary of the relationship between cfDNA and the severity of preeclampsia^*^.

**Study/Author (year)**	**Sample size**	**Detection method**	**Main findings**	**Conclusions**
Teodora et al. (2021)	20PE/22 healthy pregnant women	Total cfDNA (Qubit fluorometric assay)	Total cfDNA in the PE group was >10 times higher than controls (1,235 vs. 106.5 pg/μl, *P* < 0.001)	Total cfDNA levels correlate positively with PE severity (19).
Lorena et al. (2021)	88GH/91PE/98 healthy pregnant women	Total cfDNA (Quant-iT™ PicoGreen dsDNA assay)	Total cfDNA levels in GH and PE (197.0 and 174.2 ng/mL, respectively) were higher than in healthy pregnancies (140.5 ng/ml; all *P* < 0.0001)	Total cfDNA is elevated in HDP pregnancies (both male and female fetuses), with higher levels in severe cases (15).
Marie et al. (2023)	166PE/332 healthy pregnant women	cfDNA methylation analysis	A methylation-based model identified 72% of early-onset PE cases with 80% specificity.	cfDNA methylation analysis is a promising tool for pre-symptomatic PE risk assessment (9).
Marialuigia et al. (2023)	25PE/422 healthy pregnant women	Whole-genome bisulfite sequencing	Women with HDP showed similar cfDNA methylation patterns in the first trimester (11–14 weeks).	Maternal cardiovascular susceptibility to HDP may be detectable early via cfDNA methylation, enabling personalized screening (10).

^*^Data sources from references (8, 9, 14, 18).

## 3 The central role of cfDNA in the pathogenesis of preeclampsia: bridging immune activation and metabolic dysregulation

The core pathophysiology of preeclampsia involves systemic maternal inflammation and endothelial damage triggered by placental dysfunction. Recent studies indicate that circulating cell-free DNA (cfDNA), particularly placental-derived fetal cfDNA (cffDNA), not merely serves as a biomarker of disease severity but functions as a culprit damage-associated molecular pattern (DAMP) molecule that drives the immunoinflammatory cascade and metabolic dysregulation.

### 3.1 Mechanisms and biomolecular features of cfDNA as a DAMP

Placentas in preeclampsia commonly exhibit inadequate perfusion and ischemia-hypoxia, leading to exacerbated trophoblast cell apoptosis and/or necrosis. This process releases substantial amounts of cfDNA/cffDNA exhibiting DAMP properties. Fragmentation profile: placenta-derived cfDNA in PE patients shows significantly shortened fragments (typically < 150 bp), resembling DNA released from apoptotic bodies and facilitating immune recognition. Hypomethylation status: in preeclampsia, the free circulating cell-free fetal DNA in maternal blood is hypomethylated, indicating that methyl groups are added to cytosine-guanine (CpG) residues in the DNA molecule. Hypomethylated CpG motifs structurally mimic bacterial DNA. Mitochondrial DNA (mtDNA) enrichment: in preeclamptic placentas associated with pregnancy, there is an increased apoptosis of trophoblasts, resulting in the release of circulating fetal DNA containing mitochondrial DNA (mtDNA) (25). MtDNA similarly contains abundant unmethylated CpG islands and demonstrates striking structural similarity to bacterial DNA.

### 3.2 DAMP recognition receptor-mediated immune-inflammatory pathway activation

Elevated levels of cfDNA/cffDNA/mtDNA in maternal circulation act as DAMPs, sensed by pattern recognition receptors (PRRs) that trigger a robust sterile inflammatory response—a hallmark of PE pathophysiology. The Toll-like receptor 9 (TLR9) pathway plays a central role: as the key receptor for unmethylated CpG DNA motifs, TLR9 demonstrates significantly upregulated expression in placental tissue and peripheral blood monocytes from preeclampsia patients. Serological analysis revealed that phosphorylation levels of MyD88 and NF-κB, key downstream molecules of the TLR9 signaling pathway, were significantly elevated in peripheral blood mononuclear cells (PBMCs) from preeclamptic patients (26). Activated NF-κB translocates to the nucleus, driving the transcription and expression of multiple pro-inflammatory cytokines (e.g., TNF-α, IL-6, IL-8) and type I interferons (e.g., IFN-β). In addition, the number of extracellular vesicles (EVs) in the placentas of preeclamptic patients was approximately twofold higher than that in normal pregnancies. These EVs were enriched in mitochondrial DNA (mtDNA) and nucleosomal fragments, and may exacerbate local placental inflammation via the TLR9/NF-κB axis (27). Key experimental evidence supports this mechanism that trophoblast cells treated with preeclamptic serum exhibited significantly increased mRNA expression of TLR9 and IFN-β (28). Treatment with genomic DNA removal agents significantly inhibited the secretion of IL-6 and IL-8 induced by both normal and preeclamptic serum. Beck et al. (29) proposed that cfDNA has pro-inflammatory effects, cfDNA injection provokes IL-6 production in mice via TLR9. When both cfDNA and the TLR9 inhibitor chloroquine were administered to mice, the pregnancy outcomes improved (30).

Cytosolic DNA-Sensing Receptors Pathway (AIM2/IFI16): at the placental level, protein expression of AIM2 and IFI16 is significantly elevated in placentas from preeclamptic patients (31). This recognition triggers inflammasome assembly, activating caspase-1. Active caspase-1 then cleaves pro-IL-1β and pro-IL-18 into their mature, highly pro-inflammatory forms, while concurrently suppressing pro-angiogenic factors like PlGF, thereby exacerbating PE symptoms.

### 3.3 Pathological effects of the immunoinflammatory cascade

CfDNA activates both TLR9 and cytosolic DNA-sensing pathways, collectively driving exaggerated inflammatory responses locally (placenta) and systemically. The resulting proinflammatory cytokine storm: Characterized by massive release of TNF-α, IL-6, IL-8, IL-1β, IL-18, IFN-β, etc.—inflicts direct cytotoxic effects on placental trophoblast cells and maternal vascular endothelial cells. Release of Anti-angiogenic Factors: at the RNA level, the mRNA expression of IL-6 and IL-8 is significantly elevated in trophoblast cells treated with preeclamptic serum compared to those treated with serum from normal pregnancies. However, there is no significant difference in mRNA expression of Eng and Flt1 between the two groups. At the protein level, trophoblast cells stimulated with preeclamptic serum exhibit significantly increased secretion of soluble endoglin (sEng) and soluble fms-like tyrosine kinase-1 (sFlt-1) (32, 33). However, the protein levels of IL-6 and IL-8 remained below the detection threshold in both preeclamptic and normal serum-treated groups. Notably, no significant differences were observed in the expression of Eng and Flt1 genes in placental tissues from preeclamptic patients compared to controls, despite markedly elevated serum levels of sEng and sFlt-1 (34, 35). These findings suggest that preeclamptic serum exacerbates placental dysfunction primarily by enhancing the secretion of anti-angiogenic factors (sEng and sFlt-1) from trophoblast cells, rather than by upregulating their gene expression. Endothelial Injury and Systemic Inflammation: proinflammatory cytokines and anti-angiogenic factors act synergistically to damage maternal vascular endothelium, leading to systemic vasoconstriction, hypertension, coagulation activation, and multi-organ hypoperfusion—the cardinal clinical manifestations of preeclampsia.

Research has reported that inflammatory cells in preeclamptic patients, including neutrophils and monocytes, are activated and secrete large amounts of inflammatory cytokines. Even in the absence of microbial infection, inflammation associated with preeclampsia occurs, representing a form of sterile inflammation. In preeclamptic patients, the number of trophoblast-derived extracellular vesicles entering the maternal bloodstream significantly increases. These extracellular vesicles contain various factors, including DNA, RNA, lipids, and proteins, acting as damage-associated molecular patterns (DAMPs). In fact, extracellular vesicles can induce sterile inflammation and preeclampsia-like features in mouse placentas. Recent studies indicate that cfDNA, considered a product of apoptosis and/or necrosis, functions as a DAMP and is associated with various inflammatory diseases (27). During pregnancy, the total amount of cfDNA in maternal blood significantly increases, and elevated cfDNA levels are significantly correlated with pregnancy complications. Empirical findings are detailed in Table 2.

**Table 2:** Summary of the immune relationship between cfDNA and preeclampsia^*^.

**Study/Author (year)**	**Sample size**	**Detection method**	**Immune-related markers**	**Main findings**	**Conclusions**
Ane Cecilie et al. (2024)	75PE/37 healthy pregnant women	SomaScan assay	sFlt-1, Endothelin, PlGF	Higher levels of sFlt-1 and endothelin-1 in PE, especially in early-onset cases.	Maternal sFlt-1 and endothelin-1 levels increase in PE, with the highest levels in early-onset cases (34).
Ruby et al. (2023)	194 PE/194 healthy pregnant women	Immunohistochemistry	TLR-4, HMGB1, NF-κB, IκBα	Upregulation of TLR pathway (TLR-4, HMGB1, NF-κB, IκBα) and hypoxia markers in PE placentas.	In preeclampsia, the placenta exhibits aberrant activation of both the TLR pathway (pro-inflammatory) and the hypoxia pathway (hypoxic stress). These two pathways are interconnected and form a positive feedback loop, exacerbating placental injury (28).
Dorota et al. (2022)	35PE/45 healthy pregnant women	Immunoenzymatic assay	IL-17, PlGF, sENG	Preeclampsia patients showed markedly higher sENG levels than healthy third-trimester controls (11.47 ± 4.65 vs. 5.68 ± 2.78 ng/ml, *P* < 0.01).	sENG is elevated in PE pregnancies (33).
Jiang et al. (2022)	14PE/7 rats	Immunohistochemistry, Western blot, transcriptomics	NF-κB, LPS, Bax/Bcl-2	Transcriptomics revealed NF-κB downregulation in LPS + ghrelin-treated groups.	Ghrelin ameliorates placental trophoblast migration and apoptosis by downregulating the NF-κB signaling pathway (26).

^*^Data sources from references (25, 26, 29, 35).

### 3.4 Association between cfDNA and metabolic dysregulation

The pathogenesis of preeclampsia further involves aberrations in metabolic pathways, in which the release and sensing of cfDNA may play interrelated roles: mitochondrial dysfunction and the release of mtDNA contribute to this process: placental ischemia and hypoxia not only promote cell death, leading to the release of cfDNA and mtDNA, but also reflect an underlying disruption in mitochondrial bioenergetics. Circulating cell-free mitochondrial DNA (ccf-mtDNA) is considered a DAMP molecule that triggers immune responses by activating the pattern recognition receptor Toll-like receptor 9 (TLR9). Compared to gestational age–matched healthy pregnant individuals, those with preeclampsia exhibit aberrations in circulating DNA dynamics, including elevated levels of ccf-mtDNA and compromised DNA clearance mechanisms (36).

Notably, this mechanism of immune activation mediated by mtDNA is not unique to PE. In sepsis, a systemic inflammatory response syndrome secondary to bacterial infection, mitochondrial dysfunction and the subsequent release of mtDNA are also established as central to its pathogenesis (37). Here, reactive oxygen species (ROS) and reactive nitrogen species can induce functional impairment in multiple organelles, including mitochondria, thereby amplifying the inflammatory cascade. Furthermore, studies suggest that variations in mitochondrial DNA haplogroups may influence the severity and progression of sepsis. This analogy provides strong supportive evidence for understanding the similar role of mtDNA in amplifying inflammation in preeclampsia.

Aberrant Expression of Metabolism-Associated Genes: canonical preeclampsia-associated genes—previously implicated in metabolic regulation, hypoxia, and angiogenesis—include LEP, HK2, FSTL3, FLT1, ENG, TMEM45A, ARHGEF4, and HTRA1. These genes have consistently been shown to be upregulated in preeclamptic placentas compared with normotensive controls across diverse ancestral backgrounds. In the present study, RNA sequencing of placental tissue from patients with severe PE (sPE) of African, Asian, and European ancestry confirmed the sustained upregulation of these canonical genes across all cohorts. Among them, HK2, FSTL3, LEP, and FLT1 exhibited the most pronounced increases. Hexokinase 2 (HK2), which plays a critical role in glucose metabolism, has been frequently reported to be dysregulated in PE (38). Leptin (LEP), a secreted adipokine, systemically regulates energy homeostasis, neuroendocrine signaling, and cytoplasmic metabolic processes. Elevated early-pregnancy leptin levels are a recurrent observation in PE patients, and exogenous leptin administration in murine models recapitulates key clinical features of preeclampsia (39).

The potential role of the cGAS-STING pathway: cytosolic DNA (including cfDNA/mtDNA) is recognized by cyclic GMP-AMP synthase (cGAS), catalyzing the production of the second messenger cyclic GMP-AMP (cGAMP). In pregnancies complicated by PE, increased cGAMP levels are associated with elevated circulating natriuretic peptides. Transfected cytosolic DNA triggers cGAMP production, which binds to the endoplasmic reticulum-resident protein STING and subsequently activates interferon regulatory factor 3 (IRF3) and interferon-beta (IFN-β). As a nucleotide second messenger belonging to the family of cyclic dinucleotides, cGAMP is capable of forming unique2′5′ phosphodiester linkages. Taken together, it is plausible that circulating fetal DNA in PE behaves in a manner similar to bacterial or mitochondrial DNA, potentially activating the cGAS–STING pathway and inducing inflammation (40). This process represents another potential pathway through which cfDNA drives inflammation, meriting further research. The interplay mechanisms between cfDNA and placental metabolic perturbations, including bioenergetic failure and oxidative stress, along with their clinical implications, are systematically collated in Table 3.

**Table 3:** Summary of the metabolic relationship between cfDNA and preeclampsia^*^.

**Study/Author (year)**	**Sample size**	**Detection method**	**Metabolic markers**	**Main findings**	**Conclusions**
SpencerC et al. (2022)	19PE/19 healthy pregnant women	ccfDNA (qPCR), ELISA	ccf-mtDNA, cf-nDNA, DNase I, TLR9	Lower ccf-mtDNA in PE (*P* ≤ 0.02), but no difference in PBMC mtDNA copy number (*P* > 0.05).	PE is associated with abnormal cfDNA dynamics, including reduced ccf-mtDNA and impaired DNA clearance (36).
Deeksha et al. (2021)	17PE/15 healthy pregnant women	qRT-PCR	MT-ND1, NADH dehydrogenase 1	Higher mtDNA copies in PE (median: 24.32 vs. 20.32), especially in early-onset cases (28.06).	Early-onset PE may involve severe mitochondrial damage, elevating mtDNA copies as a compensatory response (25).
Omonigho et al. (2023)	50PE/73 healthy pregnant women	RNA sequencing	LEP, HK2, FSTL3, FLT1, ENG, HTRA1	Upregulated HK2 (*P* < 0.01) and elevated placental cfDNA in severe PE, suggesting enhanced glycolysis.	Classic PE genes (e.g., LEP, FSTL3, HK2, FLT1) are highly upregulated in PE (38).
Huang et al. (2021)	78PE/95 healthy pregnant women	ELISA	LEP, Cer	Lep levels were higher in PE compared with non-PE placentas (*P* < 0.05)	PE placentas exhibit significantly elevated Lep expression (39).

^*^Data sources from references (36, 37, 39, 40).

## 4 Ntegrated mechanistic model of preeclampsia pathogenesis

Placental ischemia-hypoxia initiates a cascade beginning with increased trophoblast apoptosis and necrosis. This triggers the release of cfDNA/cffDNA exhibiting hallmark damage-associated molecular patterns (DAMPs), including hypomethylated CpG, short fragments, and mitochondrial DNA enrichment. Such DNA fragments are recognized by pattern recognition receptors (PRRs) such as TLR9, AIM2, IFI16, and potentially cGAS. Subsequent activation of downstream signaling pathways—including MyD88/NF-κB, inflammasomes, and STING—induces pro-inflammatory cytokine storms (TNF-α, IL-6, IL-8, IL-1β, IL-18, IFN-β) and promotes the release of anti-angiogenic factors (sFlt-1, sEng). These events collectively damage placental trophoblasts and maternal vascular endothelium, ultimately manifesting as hypertension, proteinuria, and multi-system injury characteristic of preeclampsia.

**Figure 1:**
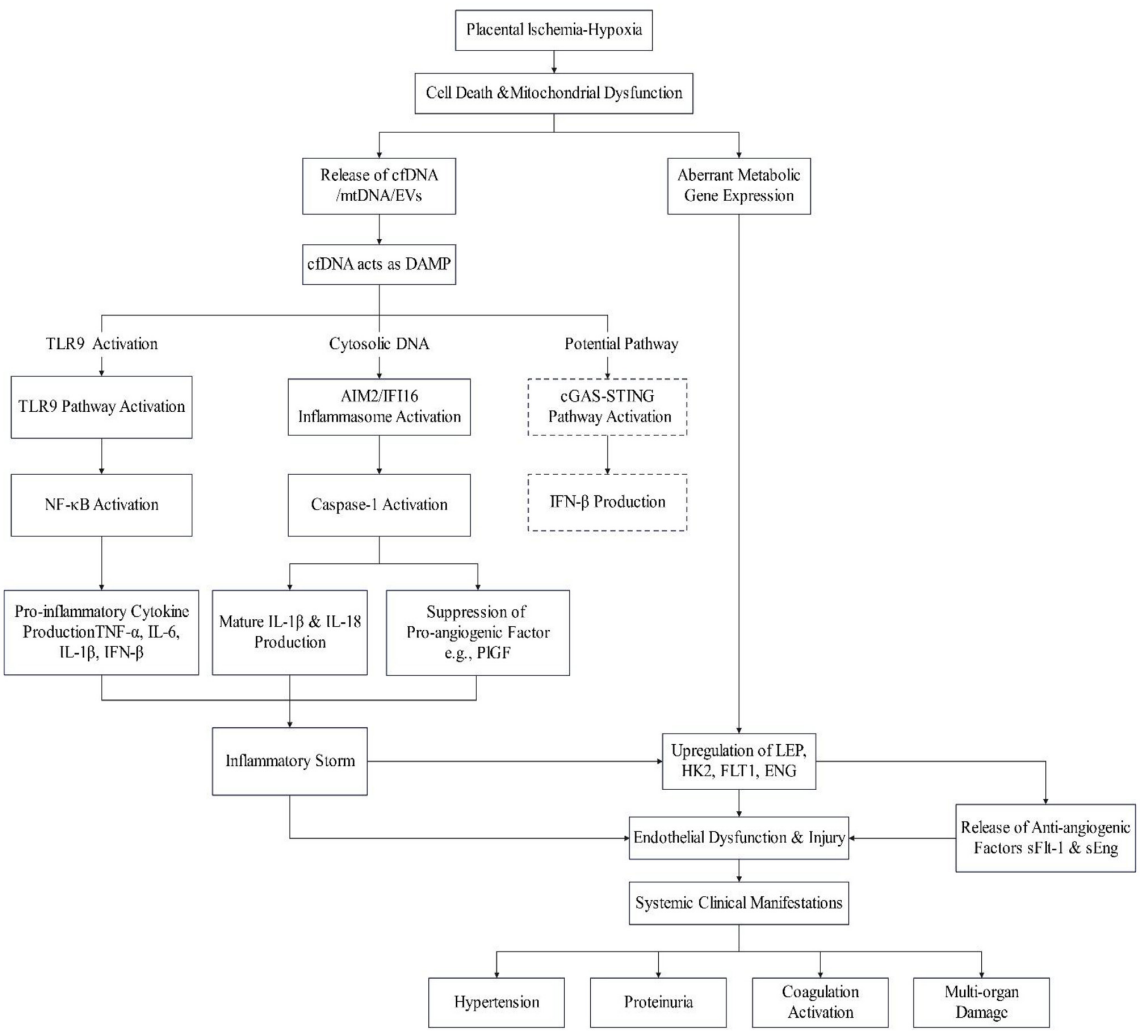
The central role of cfDNA in driving inflammation and endothelial dysfunction in preeclampsia.

Concurrently, hypoxic and inflammatory microenvironments feedback to dysregulate placental metabolic gene expression (e.g., LEP, HK2), exacerbating metabolic imbalance. Critically, cfDNA serves as a linchpin molecule integrating four key pathological axes: placental injury, immune-inflammatory activation, endothelial dysfunction, and metabolic dysregulation, thereby constituting a core pathophysiological mechanism in preeclampsia. The integrated mechanisms underlying the pathogenesis of preeclampsia are shown in Figure 1.

## 5 Clinical feasibility and validation status of multi-omics integrated predictive models

Integrated multi-omics models combining cfDNA fragmentomics, proteomics, and metabolomics significantly enhance early preeclampsia (PE) risk stratification by concurrently capturing multidimensional signals of placental hypoxia, inflammatory activation, and metabolic dysregulation. Technically, a single maternal plasma sample enables compatible analysis of cfDNA fragmentation, proteins (e.g., sFlt-1/PlGF), and metabolites (e.g., leucine), with standardized workflows for next-generation sequencing (NGS) and liquid chromatography–tandem mass spectrometry (LC-MS/MS) validated in large cohorts (e.g., SPREE study). Retrospective studies demonstrate superior predictive performance of cfDNA methylation combined with proteomics for early-onset PE (9), while the prospective PREMOM trial further reveals that integrating cfDNA fragmentomics and metabolomics increases the positive predictive value (PPV) at 16 gestational weeks from 42% (conventional models) to 67% (21). Nevertheless, real-world implementation faces critical challenges: population-specific calibration algorithms are required to address baseline omics heterogeneity induced by maternal ethnicity and comorbidities (e.g., chronic hypertension); low cfDNA abundance in early gestation (< 12 weeks) compromises dynamic monitoring accuracy, necessitating compensation via ultrasound biomarkers; additionally, although multi-omics screening reduced preterm birth by 24% in the NHS Scotland trial, its 30% cost increase and lack of long-term health economic evaluations hinder widespread adoption. Future translation should prioritize: developing microfluidic point-of-care systems for ≤ 4-h reporting; establishing international multi-omics data registries (e.g., IPD Meta-analysis Consortium) to accelerate model refinement; and defining cost-containment targets for resource-limited settings.

## 6 Conclusion

The advancement of cfDNA analysis is reshaping prenatal medicine practice. This review highlights that plasma cfDNA concentration is significantly elevated (3–5 fold) in preeclampsia (PE) patients and is strongly associated with placental hypoxia-induced trophoblast apoptosis, underscoring its considerable potential as a pathological biomarker for PE. Multi-parameter models based on cfDNA—integrating fetal fraction, fragmentomics, and placenta-specific methylation markers—outperform traditional serological biomarkers (e.g., PlGF and sFlt-1) in enhancing early PE prediction accuracy. The non-invasive nature of cfDNA analysis makes it particularly suitable for large-scale screening; however, clinical translation requires defining the optimal gestational window. Existing evidence suggests the second trimester (16–20 weeks) is a feasible and effective window for cfDNA-based risk stratification, while its first-trimester (12–14 weeks) potential hinges on further validation of ultra-sensitive technologies, such as targeted methylation sequencing—a key focus for future prospective multicenter studies.

It is essential to recognize that key scientific bottlenecks still exist in current research. First, the short half-life of cfDNA has yet to establish a standardized optimal detection window, which contrasts with the methodological differences seen in long half-life protein biomarkers. Second, there is a lack of uniform quality control standards for comparing results across different detection platforms, which may affect the reproducibility of multi-center studies. More importantly, the causal relationship between elevated cfDNA levels and preeclampsia remains unclear: whether it is passive release due to placental ischemia or active secretion triggered by maternal inflammation. This ongoing debate regarding the underlying mechanism directly impacts the choice of intervention strategies.

Future clinical research should focus on: developing integrated models that combine cfDNA with other clinical parameters (e.g., uterine artery Doppler) to improve first-trimester predictive performance; conducting large-scale, multi-ethnic prospective cohorts to rigorously validate screening efficacy and cost-effectiveness across early and mid-pregnancy; and establishing clinical pathways for managing incidental findings, such as maternal malignancy. Ultimately, translating cfDNA from a research tool into routine clinical screening will be pivotal for enabling early PE identification and personalized intervention, profoundly transforming prenatal care practice.

## References

[1] Dimitriadis E Rolnik DL Zhou W Estrada-Gutierrez G Koga K Francisco RPV. Pre-eclampsia. Nat Rev Dis Primers. (2023) 9:8. 10.1038/s41572-023-00417-6 36797292

[2] Chappell LC Cluver CA Kingdom J Tong S. Pre-eclampsia. Lancet. (2021) 398:341–54. 10.1016/S0140-6736(20)32335-7 34051884

[3] Qi J Wu B Chen X Wei W Yao X. Diagnostic biomolecules and combination therapy for pre-eclampsia. Reprod Biol Endocrinol. (2022) 20:136. 10.1186/s12958-022-01003-3 36068569 PMC9446775

[4] Hu Z Chen H Long Y Li P Gu Y. The main sources of circulating cell-free DNA: apoptosis, necrosis and active secretion. Crit Rev Oncol Hematol. (2021) 157:103166. 10.1016/j.critrevonc.2020.103166 33254039

[5] Oellerich M Sherwood K Keown P Schütz E Beck J Stegbauer J. Liquid biopsies: donor-derived cell-free DNA for the detection of kidney allograft injury. Nat Rev Nephrol. (2021) 17:591–603. 10.1038/s41581-021-00428-0 34031575

[6] Yuen N Lemaire M Wilson SL. Cell-free placental DNA: what do we really know? PLoS Genet. (2024) 20:e1011484. 10.1371/journal.pgen.1011484 39652523 PMC11627368

[7] Moufarrej MN Bianchi DW Shaw GM Stevenson DK Quake SR. Noninvasive prenatal testing using circulating DNA and RNA: advances, challenges, and possibilities. Annu Rev Biomed Data Sci. (2023) 6:397–418. 10.1146/annurev-biodatasci-020722-094144 37196360 PMC10528197

[8] Norton ME MacPherson C Demko Z Egbert M Malone F Wapner RJ. Obstetrical, perinatal, and genetic outcomes associated with nonreportable prenatal cell-free DNA screening results. Am J Obstet Gynecol. (2023) 229:300.e1–e9. 10.1016/j.ajog.2023.03.026 36965866

[9] De Borre M Che H Yu Q Lannoo L De Ridder K Vancoillie L. Cell-free DNA methylome analysis for early preeclampsia prediction. Nat Med. (2023) 29:2206–15. 10.1038/s41591-023-02510-5 37640858

[10] Spinelli M Zdanowicz JA Keller I Nicholson P Raio L Amylidi-Mohr S. Hypertensive disorders of pregnancy share common cfDNA methylation profiles. Sci Rep. (2022) 12:19837. 10.1038/s41598-022-24348-6 36400896 PMC9674847

[11] Bokuda K Ichihara A. Preeclampsia up to date—What's going on? Hypertens Res. (2023) 46:1900–7. 10.1038/s41440-023-01323-w 37268721 PMC10235860

[12] Meng Y Meng Y Li L Li Y He J Shan Y. The role of DNA methylation in placental development and its implications for preeclampsia. Front Cell Dev Biol. (2024) 12:1494072. 10.3389/fcell.2024.1494072 39691449 PMC11649665

[13] Jia R Zhu J Zhang F Sun Y Zhang B Du Y. Exploration of cfDNA landscape in NIPT and clinical utilities of cfDNA based gene expression inference in prenatal diagnostics. Front Genet. (2025) 16:1527884. 10.3389/fgene.2025.1527884 40061129 PMC11885227

[14] Zhou Z Ma ML Chan RWY Lam WKJ Peng W Gai W. Fragmentation landscape of cell-free DNA revealed by deconvolutional analysis of end motifs. Proc Natl Acad Sci U S A. (2023) 120:e2220982120. 10.1073/pnas.2220982120 37075072 PMC10151549

[15] Amaral LM Sandrim VC Kutcher ME Spradley FT Cavalli RC Tanus-Santos JE. Circulating total cell-free DNA levels are increased in hypertensive disorders of pregnancy and associated with prohypertensive factors and adverse clinical outcomes. Int J Mol Sci. (2021) 22:564. 10.3390/ijms22020564 33429954 PMC7826953

[16] Del Vecchio G Li Q Li W Thamotharan S Tosevska A Morselli M. Cell-free DNA methylation and transcriptomic signature prediction of pregnancies with adverse outcomes. Epigenetics. (2021) 16:642–61. 10.1080/15592294.2020.1816774 33045922 PMC8143248

[17] Zaki-Dizaji M Shafiee A Kohandel Gargari O Fathi H Heidary Z. Maternal and fetal factors affecting cell-free fetal DNA (cffDNA) fraction: a systematic review. J Reprod Infertil. (2023) 24:219–31. 10.18502/jri.v24i4.14149 38164433 PMC10757682

[18] Jayashankar SS Nasaruddin ML Hassan MF Dasrilsyah RA Shafiee MN Ismail NAS. Non-invasive prenatal testing (NIPT): reliability, challenges, and future directions. Diagnostics. (2023) 13:2570. 10.3390/diagnostics13152570 37568933 PMC10417786

[19] Kolarova TR Gammill HS Nelson JL Lockwood CM Shree R. At preeclampsia diagnosis, total cell-free DNA concentration is elevated and correlates with disease severity. J Am Heart Assoc. (2021) 10:e021477. 10.1161/JAHA.121.021477 34310191 PMC8475684

[20] Tarca AL Taran A Romero R Jung E Paredes C Bhatti G. Prediction of preeclampsia throughout gestation with maternal characteristics and biophysical and biochemical markers: a longitudinal study. Am J Obstet Gynecol. (2022) 226:126.e1–e22. 10.1016/j.ajog.2021.01.020 34998477 PMC8749051

[21] MacDonald TM Walker SP Hannan NJ Tong S Kaitu'u-Lino TJ. Clinical tools and biomarkers to predict preeclampsia. EBioMedicine. (2022) 75:103780. 10.1016/j.ebiom.2021.103780 34954654 PMC8718967

[22] Martin K Dar P MacPherson C Egbert M Demko Z Parmar S. Performance of prenatal cfDNA screening for sex chromosomes. Genet Med. (2023) 25:100879. 10.1016/j.gim.2023.100879 37154148

[23] Shear MA Swanson K Garg R Jelin AC Boscardin J Norton ME. A systematic review and meta-analysis of cell-free DNA testing for detection of fetal sex chromosome aneuploidy. Prenat Diagn. (2023) 43:133–43. 10.1002/pd.6298 36588186 PMC10268789

[24] Iannaccone A Reisch B Mavarani L Darkwah Oppong M Kimmig R Mach P. Soluble endoglin versus sFlt-1/PlGF ratio: detection of preeclampsia, HELLP syndrome, and FGR in a high-risk cohort. Hypertens Pregnancy. (2022) 41:159–72. 10.1080/10641955.2022.2066119 35475405

[25] Pandey D Yevale A Naha R Kuthethur R Chakrabarty S Satyamoorthy K. Mitochondrial DNA copy number variation - a potential biomarker for early onset preeclampsia. Pregnancy Hypertens. (2021) 23:1–4. 10.1016/j.preghy.2020.10.002 33160129

[26] Shen J Hu N Wang Z Yang L Chen R Zhang L. Ghrelin alleviates placental dysfunction by down-regulating NF-κB phosphorylation in LPS-induced rat model of preeclampsia. Eur J Pharmacol. (2024) 972:176569. 10.1016/j.ejphar.2024.176569 38593930

[27] Roh JS Sohn DH. Damage-associated molecular patterns in inflammatory diseases. Immune Netw. (2018) 18:e27. 10.4110/in.2018.18.e27 30181915 PMC6117512

[28] Aggarwal R Jain AK Mehta V Rath G. Amalgamation of toll-like receptor and hypoxic signaling in etiology of preeclampsia. Appl Immunohistochem Mol Morphol. (2023) 31:429–37. 10.1097/PAI.0000000000001129 37249078

[29] Beck S Buhimschi IA Summerfield TL Ackerman WE Guzeloglu-Kayisli O Kayisli UA. Toll-like receptor 9, maternal cell-free DNA and myometrial cell response to CpG oligodeoxynucleotide stimulation. Am J Reprod Immunol. (2019) 81:e13100. 10.1111/aji.13100 30758898 PMC6453711

[30] Choi M Hwang JR Sung JH Byun N Seok YS Cho GJ. Hydroxychloroquine reduces hypertension and soluble fms-like kinase-1 in a Nω-nitro-l-arginine methyl ester-induced preeclampsia rat model. J Hypertens. (2022) 40:2459–68. 10.1097/HJH.0000000000003279 36321404

[31] Fan Z Chen R Yin W Xie X Wang S Hao C. Effects of AIM2 and IFI16 on infectious diseases and inflammation. Viral Immunol. (2023) 36:438–48. 10.1089/vim.2023.0044 37585649

[32] Garrido-Giménez C Cruz-Lemini M Álvarez FV Nan MN Carretero F Fernández-Oliva A. Predictive model for preeclampsia combining sFlt-1, PlGF, NT-proBNP, and uric acid as biomarkers. J Clin Med. (2023) 12:431. 10.3390/jcm12020431 36675361 PMC9866466

[33] Darmochwal-Kolarz D Chara A. The association of IL-17 and PlGF/sENG ratio in pre-eclampsia and adverse pregnancy outcomes. Int J Environ Res Public Health. (2022) 20:768. 10.3390/ijerph20010768 36613090 PMC9819392

[34] Westerberg AC Degnes ML Andresen IJ Roland MCP Michelsen TM. Angiogenic and vasoactive proteins in the maternal-fetal interface in healthy pregnancies and preeclampsia. Am J Obstet Gynecol. (2024) 231:550.e1–e22. 10.1016/j.ajog.2024.03.012 38494070

[35] Mitranovici MI Chiorean DM Moraru R Moraru L Caravia L Tiron AT. Understanding the pathophysiology of preeclampsia: exploring the role of antiphospholipid antibodies and future directions. J Clin Med. (2024) 13:2668. 10.3390/jcm13092668 38731197 PMC11084819

[36] Cushen SC Ricci CA Bradshaw JL Silzer T Blessing A Sun J. Reduced maternal circulating cell-free mitochondrial DNA is associated with the development of preeclampsia. J Am Heart Assoc. (2022) 11:e021726. 10.1161/JAHA.121.021726 35014857 PMC9238514

[37] Filetici N Van de Velde M Roofthooft E Devroe S. Maternal sepsis. Best Pract Res Clin anaesthesiol. (2022) 36:165–77. 10.1016/j.bpa.2022.03.003 35659952

[38] Aisagbonhi O Bui T Nasamran CA St Louis H Pizzo D Meads M. High placental expression of FLT1, LEP, PHYHIP and IL3RA - in persons of African ancestry with severe preeclampsia. Placenta. (2023) 144:13–22. 10.1016/j.placenta.2023.10.008 37949031 PMC10843761

[39] Huang Q Hao S You J Yao X Li Z Schilling J. Early-pregnancy prediction of risk for pre-eclampsia using maternal blood leptin/ceramide ratio: discovery and confirmation. BMJ Open. (2021) 11:e050963. 10.1136/bmjopen-2021-050963 34824115 PMC8627403

[40] Tsuji N Agbor-Enoh S. Cell-free DNA beyond a biomarker for rejection: biological trigger of tissue injury and potential therapeutics. J Heart Lung Transpl. (2021) 40:405–13. 10.1016/j.healun.2021.03.007 33926787

